# USP22 mediates the multidrug resistance of hepatocellular carcinoma via the SIRT1/AKT/MRP1 signaling pathway

**DOI:** 10.1002/1878-0261.12067

**Published:** 2017-05-11

**Authors:** Sunbin Ling, Jie Li, Qiaonan Shan, Haojiang Dai, Di Lu, Xue Wen, Penghong Song, Haiyang Xie, Lin Zhou, Jimin Liu, Xiao Xu, Shusen Zheng

**Affiliations:** ^1^ Division of Hepatobiliary and Pancreatic Surgery Department of Surgery, Collaborative Innovation Center for Diagnosis and Treatment of Infectious Diseases the First Affiliated Hospital Zhejiang University School of Medicine Hangzhou China; ^2^ Key Laboratory of Combined Multi‐organ Transplantation Key Laboratory of Organ Transplantation Ministry of Public Health Hangzhou China; ^3^ Key Laboratory of Organ Transplantation Hangzhou Zhejiang China; ^4^ Department of Cancer Biology University of Pennsylvania Perelman School of Medicine Philadelphia PA USA; ^5^ Department of Pathology First Affiliated Hospital School of Medicine Zhejiang University China; ^6^ Department of Pathology and Molecular Medicine Faculty of Health Sciences McMaster University Hamilton Canada

**Keywords:** hepatocellular carcinoma, MRP1, multidrug resistance, SIRT1, USP22

## Abstract

Drug treatments for hepatocellular carcinoma (HCC) often fail because of multidrug resistance (MDR). The mechanisms of MDR are complex but cancer stem cells (CSCs), which are able to self‐renew and differentiate, have recently been shown to be involved. The deubiquitinating enzyme ubiquitin‐specific protease 22 (USP22) is a marker for CSCs. This study aimed to elucidate the role of USP22 in MDR of HCC and the underlying mechanisms. Using *in vitro* and *in vivo* assays, we found that modified USP22 levels were responsible for the altered drug‐resistant phenotype of BEL7402 and BEL/FU cells. Downregulation of USP22 dramatically inhibited the expression of ABCC1 (encoding MRP1) but weakly influenced ABCB1 (encoding P‐glycoprotein). Sirtuin 1 (SIRT1) was reported previously as a functional mediator of USP22 that could promote HCC cell proliferation and enhance resistance to chemotherapy. In this study, USP22 directly interacted with SIRT1 and positively regulated SIRT1 protein expression. Regulation of the expression of both USP22 and SIRT1 markedly affected the AKT pathway and MRP1 expression. Inhibition of the AKT pathway by its specific inhibitor LY294002 resulted in downregulation of MRP1. USP22 and MRP1 expression was detected in 168 clinical HCC samples by immunohistochemical staining, and a firm relationship between USP22 and MRP1 was identified. Together, these results indicate that USP22 could promote the MDR in HCC cells by activating the SIRT1/AKT/MRP1 pathway. USP22 might be a potential target, through which the MDR of HCC in clinical setting could be reversed.

**Abbreviations**
CSC: cancer stem cell
HCC: hepatocellular carcinoma
MDR: multidrug resistance
MRP1: resistance‐associated protein 1
P‐gp: P‐glycoprotein
SIRT1: sirtuin 1
USP22: ubiquitin‐specific protease 22

## 1 Introduction

Hepatocellular carcinoma (HCC) is one of the most common malignancies and results in approximately 80 000 patient deaths worldwide per year, representing the second leading cause of cancer death (Torre *et al*., 2015). The front‐line treatment for HCC is surgical resection and liver transplantation. However, most HCCs are inoperable because patients typically present at advanced stages. Even after surgical resection, the prognosis of HCC remains poor given the high recurrence rate. For advanced‐stage HCCs, systematic therapies are adopted as palliative methods, such as traditional chemotherapy and molecular targeted therapy, but most of them failed to demonstrate significant efficacy in increasing survival rates (Chan *et al*., 2016; Lin *et al*., 1997). The response rates of traditional cytotoxic chemotherapy agents, such as adriamycin, fluorouracil, cisplatin, and mitomycin to HCC, are less than 10% (Ge and Huang, 2015; Qin *et al*., 2013; Zaanan *et al*., 2013). To date, although sorafenib is the only small‐molecule inhibitor that has been approved by the U.S. Food and Drug Administration for HCC treatment, the survival benefit of the sorafenib treatment arm was modest (Cheng *et al*., 2009). Multidrug resistance (MDR) is the main reason for HCC drug therapy failure, and complex mechanisms are involved. Extensive studies have revealed that MDR is associated with the epithelial/mesenchymal transition (van Zijl *et al*., 2011), overexpression of drug efflux pumps, DNA damage repair, the hypoxia‐inducible factor 1‐α signaling pathway, and epigenetic regulation. Recent studies indicate that cancer stem cells (CSCs), which are able to self‐renew and differentiate, are involved in the mechanism of chemoresistance (Chan *et al*., 2015).

Ubiquitin‐specific protease 22 (USP22), which is described as a CSC marker (Glinsky, 2006; Zhang *et al*., 2008), belongs to the ubiquitin‐specific processing proteases (USPs), the largest subfamily of deubiquitinating enzymes (DUBs) (Nijman *et al*., 2005). USP22 is a key subunit of the Spt‐Ada‐Gcn5 acetyl transferase complex (SAGA), which removes ubiquitin from target proteins to regulate the transcription of downstream genes (Reyes‐Turcu *et al*., 2009). USP22 plays a significant role both in pathology and in physiology via interactions with different substrates (Melo‐Cardenas *et al*., 2016). USP22 could bind to TRF1 and regulate telomere length (Atanassov *et al*., 2009). USP22 deubiquitinates cyclin B1, stabilizes cyclin B1 by antagonizing proteasome‐mediated degradation, and promotes it accumulation in the nucleus (Lin *et al*., 2015; Melo‐Cardenas *et al*., 2016). Recently, USP22 was found to cooperate with GSK3β to stabilize KDM1A and further promote glioblastoma tumorigenesis (Zhou *et al*., 2016). Clinically, USP22 has been confirmed to predict tumor recurrence, metastasis, and poor survival after cancer diagnosis in several types of cancer (Glinsky *et al*., 2005; He *et al*., 2015). Our group first ensured that knockdown of USP22 can induce cell cycle arrest and inhibit cell growth in the HCC cell line HepG2 (Ling *et al*., 2012a).

Notably, sirtuin 1 (SIRT1), a member of the sirtuin family of nicotinamide adenine dinucleotide (NAD+)‐dependent class III histone deacetylases that are human homologues of yeast silent information regulator 2, controls a variety of biologic processes ranging from metabolic homeostasis, neurodegenerative diseases, and aging to cancer (Armour *et al*., 2013). Chen *et al*. (2012) demonstrated that SIRT1 was overexpressed in HCC and plays an oncogenic role in HCC by enhancing cell proliferation and resistance to chemotherapy. Interestingly, SIRT1 was identified as a mediator of acetylation of USP22 and the SAGA coactivator complex (Armour *et al*., 2013). USP22 may antagonize p53 through Sirt1 stabilization to suppress cell apoptosis under DNA damage and during embryonic development (Lin *et al*., 2012). To date, studies exploring the mechanism of USP22 influencing drug resistance of HCC is limited. In this study, we hypothesize that the CSC marker USP22 influences drug sensitivity via regulating SIRT1, which will shed new insights into the mechanisms of MDR in HCC.

## 2 Materials and methods

### 2.1 Cell cultures

The human HCC cell lines Bel7402 and Bel7402/5‐fluorouracil (BEL/FU) were purchased from KeyGen Biotech Co., Ltd (Nanjing, China). The Bel‐7402 cells were cultured in RPMI‐1640 (Gibco, Carlsbad, CA, USA) supplemented with 10% fetal bovine serum (Gibco) and 100 μg·mL^−1^ each of penicillin and streptomycin (Invitrogen, Carlsbad, CA, USA) in 5% CO_2_ at 37 °C. The BEL/FU cells were cultured as described previously (Cheng *et al*., 2013).

### 2.2 Reagents and antibodies

5‐Fluorouracil (2,4‐dihydroxy‐5‐fluoropyrimidine), methotrexate (MTX), and ADR (doxorubicin) were purchased from Sigma‐Aldrich (St. Louis, MO, USA). LY294002 was purchased from Selleck Chemicals (Houston, TX, USA). The following antibodies were used in western blot analysis and/or immunohistochemical staining. β‐Actin was from Santa Cruz Biotechnology, Inc. (Santa Cruz, CA, USA). USP22 and SIRT1 were from Abcam (Cambridge, MA, USA). AKT, phosphorylated AKT (phospho‐Ser473), phosphorylated GSK‐3β (phospho‐Ser9), cleaved PARP, and cleaved caspase‐3 were from Cell Signaling Technology, Inc. (Danvers, MA, USA). P‐glycoprotein (P‐gp) and resistance‐associated protein 1 (MRP1) were from Thermo Fisher Scientific, Inc. (Rockford, IL, USA). Goat anti‐rabbit and goat anti‐mouse IgG peroxidase‐conjugated secondary antibodies were from Thermo‐Pierce (Rockford, IL, USA). The Cell Counting Kit‐8 (CCK‐8) and the Annexin V‐FITC Apoptosis Detection Kit were purchased from KeyGen Biotech. The HistostainTM‐Plus Kits (IgG/Bio, S‐A/HRP, DAB) were purchased from Zhongshan Golden Bridge Co., Ltd. (Beijing, China).

### 2.3 *In vitro* drug cytotoxic assay

The cytotoxicity of chemotherapeutic drugs was determined by the CCK‐8 assay. Cells (5 × 10^3^) were plated in 100 mL of medium/well in 96‐well plates. After 24 h of incubation, 5‐Fu (0, 100, 200, 500, and 1000 mg·mL^−1^), MTX (0, 5, 10, 20, and 50 mg·mL^−1^), and ADR (0, 5, 10, 20, and 50 mg·mL^−1^) were added, and the CCK‐8 assay was performed 48 h later. Then, the medium was replaced with 100 μL of RPMI‐1640 and 10 μL of CCK‐8 reagent. The plates were gently shaken, and the absorbance was measured at 450 nm with an EnSpire™ 2300 Multilabel Reader (PerkinElmer, Waltham, MA, USA). Five replicates were prepared for each condition, using wells without cells as blanks. The IC_50_ values were calculated to observe the cytotoxicity of drugs.

### 2.4 Flow cytometric analysis for cell apoptosis

To evaluate the effects on induction of apoptosis, the cells were examined using the Annexin V‐APC Apoptosis Detection Kit according to the manufacturer's protocols. BEL7402 and BEL/5‐FU cells were seeded into six‐well plates (2 × 10^5^ well). The cells were trypsinized, washed with cold PBS, and suspended in PBS. Then, the cells were stained using 5 μL of Annexin V‐APC and 5 μL of propidium iodide and were incubated at 37 °C for 30 min in the dark. The stained cells were analyzed using an Accuri C6 flow cytometer (Accuri Cytometers Inc., Ann Arbor, MI, USA).

### 2.5 RT‐PCR and real‐time PCR array analysis

For RT‐PCR, the RNA isolation, PCR, and primers for amplification were as described before (Ling *et al*., 2012a). For the real‐time PCR array analysis, the Human Cancer Drug Resistance PCR Array (PAHS‐004Z) was purchased from QIAGEN (Valencia, CA, USA). Total RNA was isolated using the RNeasy Mini Kit (QIAGEN) and then reverse‐transcribed into first‐strand cDNA using the QuantiTect Reverse Transcription Kit (QIAGEN) according to the manufacturer's protocol. Real‐time PCR was performed on an ABI Prism 7500 fast real‐time PCR system (Applied Biosystems, Foster City, CA, USA) using the QuantiTect SYBR Green PCR Kit (QIAGEN). The data were analyzed in http://pcrdataanalysis.sabiosciences.com/pcr/arrayanalysis.php. The relative expression of the genes was normalized to β‐actin. The primer sequences of MRP1 (ABCC1) and β‐actin could be found in one of our previous studies (Ling *et al*., 2014b).

### 2.6 Immunoprecipitation and western blot analysis

An immunoprecipitation kit from Proteintech (Wuhan, China) was used in this study. The main procedure was conducted according to the manufacturer's protocol. Briefly, cell lysates were immunoprecipitated with an antibody against USP22. Precipitated proteins (10 μg) were prepared for western blot analysis, and the detailed procedure has been described previously (Ling *et al*., 2014a). Primary antibodies (described in the section 2.2.) were incubated at 4 °C overnight. The bands were visualized by chemiluminescence, imaged using a ChemiDoc XRS, and analyzed using Image Lab (both from Bio‐Rad, Hercules, CA, USA).

### 2.7 Intracellular ADR measurement

Cells were treated with 10 μm of ADR for 1 h, and then, the cells were cultured in drug‐free RPMI‐1640 for an additional 1 h. Next, the cells were trypsinized and harvested. Fluorescence intensity of intracellular ADR was determined by flow cytometry. The wavelengths of excitation light and emission light were 488 and 575 nm. Geomean was calculated and compared among different groups.

### 2.8 Gene knockdown using shRNA in BEL/FU cells

Transfection was performed in a six‐well plate with 50% confluent cultures. Cells were maintained in 2 mL of complete medium with 5 mg·mL^−1^ Polybrene per well and were treated with 0.5 μm control, USP22‐ or SIRT1‐specific shRNA lentiviral particles (obtained from Shandong Vigenebio Co., Ltd., Jinan, China) overnight. Then, the medium in each well was replaced with 2 mL of complete medium (without Polybrene). Next, stable clones expressing control, USP22‐ or SIRT1‐specific shRNA were selected using puromycin dihydrochloride. Then, cells were collected for gene expression assays.

### 2.9 Stable overexpression of USP22 or SIRT1 in BEL‐7402 cells

Lentiviral particles containing empty vector, USP22 vector, or SIRT1 vector were obtained from Shandong Vigenebio Co., Ltd. Cells were plated in a six‐well plate at 50% confluent cultures. Media were removed from plate wells and replaced with media supplemented with Polybrene at a final concentration of 5 μg·mL^−1^. Next, the cells were infected by adding lentiviral particles to the culture. Stable clones expressing empty vector, USP22, or SIRT1 were selected using puromycin dihydrochloride. Then, cells were collected for gene expression assays.

### 2.10 *In vivo* chemosensitivity assay

To investigate whether USP22 is related to drug sensitivity of tumor cells *in vivo*, the chemosensitivity of 5‐FU was examined in nude mice bearing tumor cell xenografts. Five‐week‐old male athymic nude mice were obtained from the Animal Facility of Zhejiang University. The mice were maintained under pathogen‐free conditions and were provided with sterilized food and water. Briefly, 5 × 10^6^ cells were injected subcutaneously into the right flank of each nude mouse. When mice exhibited palpable tumors (the tumor volume was approximately 100 mm^3^), BEL7402/mock, BEL7402/USP22, BEL/FU control shRNA, and BEL/FU‐USP22 shRNA tumor‐bearing mice were randomly divided into control and treatment groups (*n* = 6 mouse per group). The treatment groups received 30 mg·kg^−1^ 5‐FU (intraperitoneal injection) three times per week for 3 weeks, and the control groups received DMSO diluted in physiological saline. The mice were sacrificed, and the tumors were isolated. The tumor volume was detected and calculated using the following formula: volume = 1/2(length × width^2^).

### 2.11 Patient samples

Tumor samples from a total of 168 HCC patients who underwent operations at our hospital (First Affiliated Hospital, Zhejiang University School of Medicine, Zhejiang, China) between 2010 and 2014 were used in this study. This study was approved by the Medical Ethics Committee of the First Affiliated Hospital of Zhejiang University, and informed consent was obtained from all patients. These patients were diagnosed with HCC either before or after surgery. No treatment was administered to the patients before surgery. The diagnosis was confirmed by histopathological examination.

### 2.12 Immunohistochemical staining

The tumors isolated from the mice were paraffin‐embedded and cut into 10‐μm sections using a microtome cryostat (HM 500 OM; Carl Zeiss, Jena, Germany). Immunohistochemical staining was conducted according to the manufacturer's protocols for the HistostainTM‐Plus Kits. Primary antibodies (as described in the section 2.2.) were incubated at 4 °C overnight. The images were captured with a light microscope (Axiolab; Carl Zeiss), and five images/sample were prepared. The image‐pro plus 4.5 (Media Cybernetics, Silver Spring, MD, USA) software was used to analyze the staining data.

For the tumor samples from HCC patients, a tissue microarray was created from the 168 HCC samples. After histopathological examination, every sample was excised in one core with a 1.0 mm diameter on one tissue array. Immunohistochemical staining was conducted according to the manufacturer's protocols for the HistostainTM‐Plus Kits. The results were scanned by Pannoramic MIDI (3DHISTECH Ltd., Budapest, Hungary), quantified, and analyzed by Pannoramic viewer and quant center software. For each epitope, the staining score for tumor cells was recorded separately. An H‐score, which reflects the expression level of certain protein, was calculated based on the staining intensity as previously described (Azim *et al*., 2015).

### 2.13 Statistical analysis

spss 21.0 statistical software (Chicago, IL, USA) was used for the statistical analysis. Values are presented as the mean ± SD. Statistical analyses were performed using Student's *t*‐test. The analysis of multiple groups was performed by ANOVA with an appropriate *post hoc* test. Correlation analyses were performed using Pearson's test for quantitative data.

## 3 Results

### 3.1 USP22 is highly expressed in the HCC MDR cell line BEL/FU

**Figure 1:**
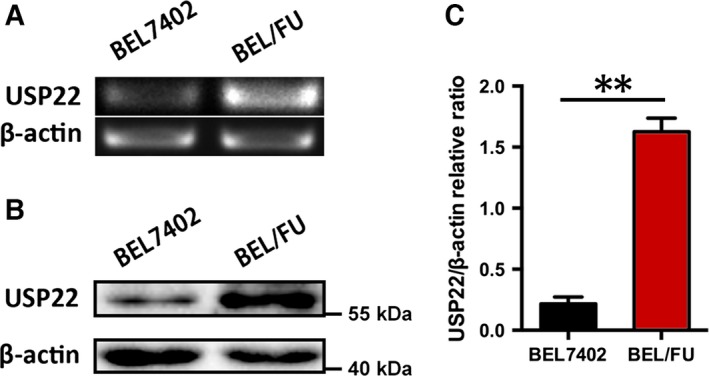
Expression of USP22 in BEL7402 and BEL/FU cells. (A) The USP22 mRNA levels were quantified by qPCR in BEL7402 and BEL/FU cells. (B) The USP22 expression was determined using western blot in BEL7402 and BEL/FU cells. (C) Band intensities are presented after semiquantification using image lab 5.0 software (Bio‐Rad) and normalized with β‐actin. These values are represented as the means ± SD from three independent experiments (**P < 0.01).

Our PCR and western blot assay results revealed drastically increased USP22 mRNA and protein expression in BEL/FU cells compared with parental BEL‐7402 cells (Fig. 1). Given that USP22 is a CSC marker, these results implied that USP22 might play a significant role in MDR in human HCC cells.

### 3.2 Modulation of USP22 effectively regulates MDR in BEL‐7402 and BEL/FU cells *in vitro* and *in vivo*

**Figure 2:**
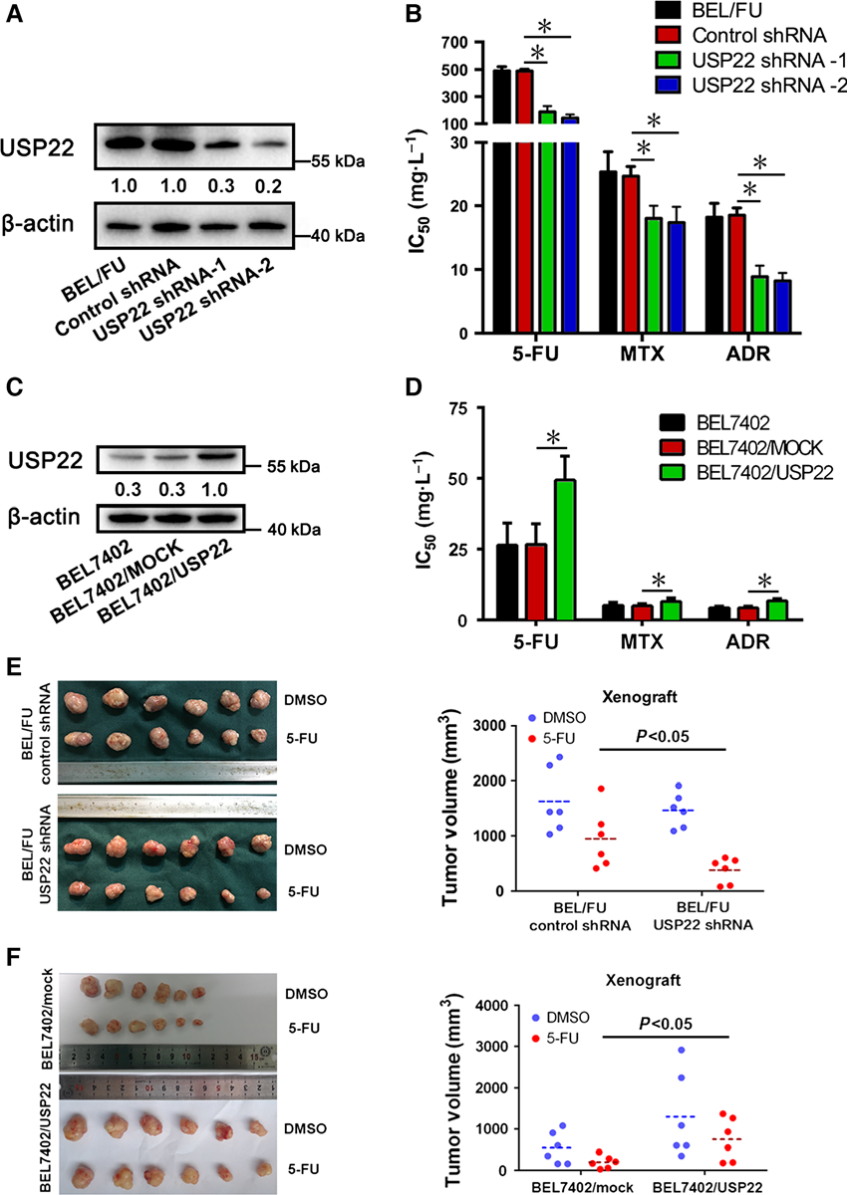
Effect of USP22 on the chemosensitivity of BEL7402 and BEL/FU cells *in vitro* and *in vivo*. (A,C) USP22 expression was monitored using western blot analysis in BEL7402 and BEL/FU cells after modulation of USP22. Band intensities were semiquantified using image lab 5.0 software and normalized with AKT or β‐actin. Values are presented as the means under the bands. (B,D) The IC_50_ values from the CCK‐8 assay were calculated to assess the sensitivity to 5‐FU, MTX, and ADR. These values are presented as means ± SD from three independent experiments (*P < 0.05). (E,F) The xenograft tumor (derived from BEL/FU control shRNA, BEL/FU USP22 shRNA, BEL7402/mock, or BEL7402/USP22 cells) volumes were measured, and the data were quantified and presented (*P < 0.05).

Given the significantly increased expression of USP22 in BEL/FU cells (Fig. 1), we silenced the USP22 gene by shRNA to elucidate the implication of USP22 in the chemosensitivity of BEL/FU cells *in vitro* and *in vivo*. As shown in Fig. 2A,B, the USP22 expression level was significantly reduced in BEL/FU cells treated with USP22 shRNA. The IC50 was calculated using the CCK‐8 assay, and silencing of USP22 significantly increased the chemosensitivity of BEL/FU cells to 5‐FU, MTX, and ADR. We also used a nude mouse model bearing BEL/FU control shRNA cell and BEL/FU control USP22 shRNA cell xenografts to elucidate the implication of USP22 in the chemosensitivity of BEL/FU cells to 5‐FU. As noted in Fig. 2E, no significant difference between BEL/FU control shRNA cells treated with DMSO and BEL/FU USP22 shRNA treated with DMSO (1627 ± 623 mm^3^ vs 1467 ± 329 mm^3^, *P* > 0.05) was observed. Remarkably, silencing USP22 reduced the tumor volume of BEL/FU cells treated with 5‐FU (BEL/FU control shRNA cells treated with 5‐FU vs BEL/FU USP22 shRNA treated with 5‐FU, 945 ± 545 mm^3^ vs 372 ± 228 mm^3^, *P *< 0.05). The *in vivo* assay results were consistent with the *in vitro* assay results.

Moreover, lentiviral vector‐mediated overexpression of USP22 protein was performed in BEL‐7402 cells. As shown in Fig. 2C,D, overexpression of USP22 significantly decreased the chemosensitivity of BEL‐7402 cells to 5‐FU, MTX, and ADR. Similarly, the *in vivo* assay results supported the *in vitro* results (Fig. 2F). These results supported the role of USP22 as a positive modulator of chemosensitivity in HCC cells.

### 3.3 Modulation of USP22 in BEL/FU and BEL‐7402 cells regulates 5‐FU‐induced cell apoptosis and intracellular ADR concentrations

**Figure 3:**
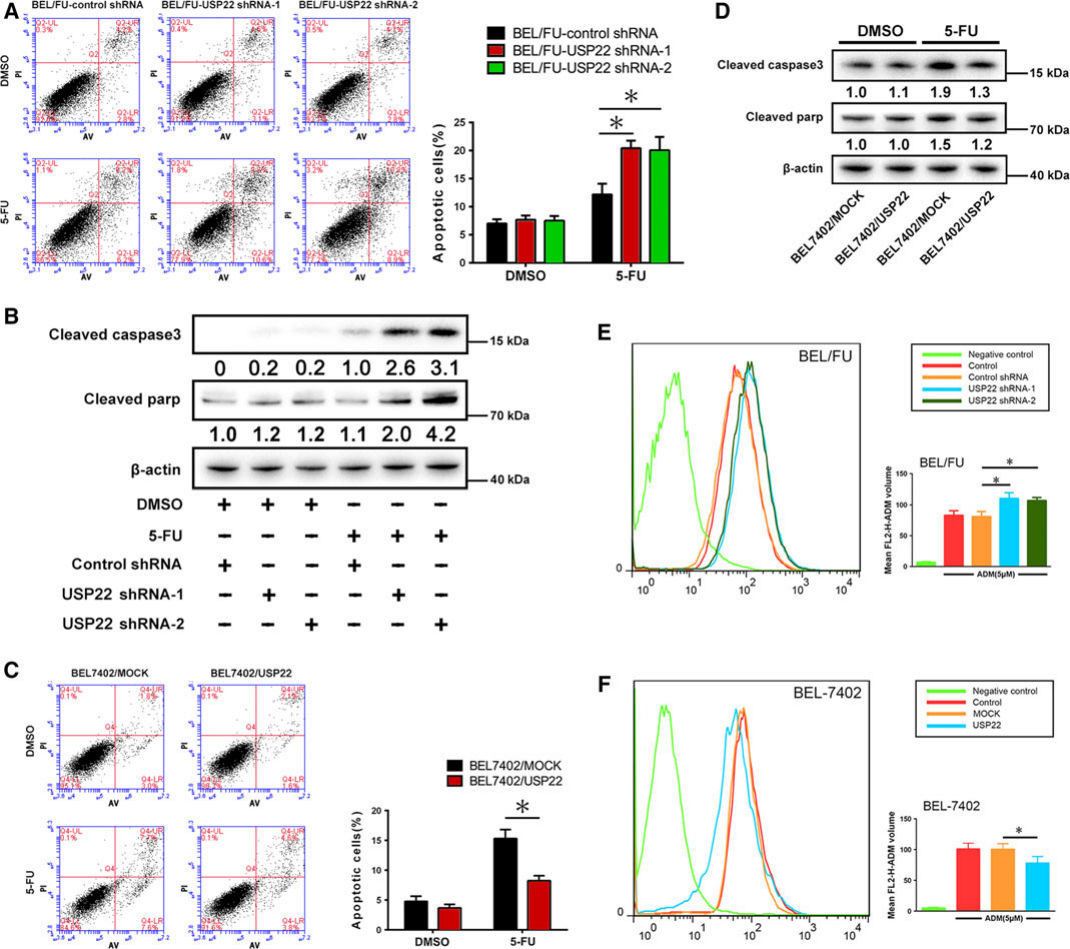
USP22 regulates cell apoptosis and intracellular ADR in BEL7402 and BEL/FU cells. (A,C) After knockdown or overexpression of USP22 in BEL/FU or BEL7402 cells, cells were examined using Annexin V/PI staining. Then, the distribution of apoptotic cells was measured by flow cytometric analysis. The percentages of apoptotic cells are presented in the bar graph (*P < 0.05). (B,D) Cleaved caspase‐3 and cleaved parp were monitored using western blot analysis in BEL7402 and BEL/FU cells. Band intensities were semiquantified using image lab 5.0 software and normalized with AKT or β‐actin. Values are represented as the means under the bands. (E,F) Intracellular ADR was measured by flow cytometric analysis. Mean FL2‐H‐ADM volume is presented in the bar graph as means ± SD from three independent experiments (*P < 0.05).

The number of apoptotic cells was examined after 48 h of 5‐FU or DMSO incubation by flow cytometric analysis. A significant increase in apoptosis was observed in BEL/FU USP22 shRNA cells with 5‐FU treatment compared with control cells, whereas no noticeable difference in the number of apoptotic cells incubated with DMSO was observed (Fig. 3A). Cleaved caspase‐3 and cleaved parp, two markers of apoptosis, were monitored to confirm 5‐FU‐induced apoptosis by western blot analysis. The levels of cleaved caspase‐3 and cleaved parp were upregulated upon inhibition of USP22 (Fig. 3B). Concomitantly, the number of apoptotic BEL‐7402/USP22 cells was reduced compared with BEL‐7402/mock cells upon treatment with 5‐FU (Fig. 3C). Cleaved caspase‐3 and cleaved parp were downregulated (Fig. 3D). To further confirm the effect of USP22 on the regulation of MDR, the fluorescence intensity of intracellular ADR was determined by flow cytometry. As shown in Fig. 3E, the concentration of intracellular ADR was increased in BEL/FU USP22 shRNA cells. In addition, overexpression of USP22 also reduced the intracellular ADR concentration in BEL7402 cells. Conclusively, these results suggested that overexpression of USP22 inhibited 5‐FU‐induced cell apoptosis and promoted ADR efflux in HCC cells.

### 3.4 PCR array results imply that downregulation of USP22 in BEL/FU cells decreases the expression of MRP1, but not P‐gp

As we previously described, USP22 silencing increased the concentration of intracellular ADR. We suggest that USP22 may have an influence on MDR proteins. A cancer drug resistance PCR array revealed that downregulation of USP22 dramatically inhibited the expression of ABCC1 (encoding MRP1) but weakly influenced ABCB1 (encoding P‐gp), as presented in Fig. S1A. The expression of P‐gp was verified by western blot analysis (Fig. S1B).

### 3.5 USP22 activates the AKT/MRP1 pathway depending on SIRT1 in HCC cells

**Figure 4:**
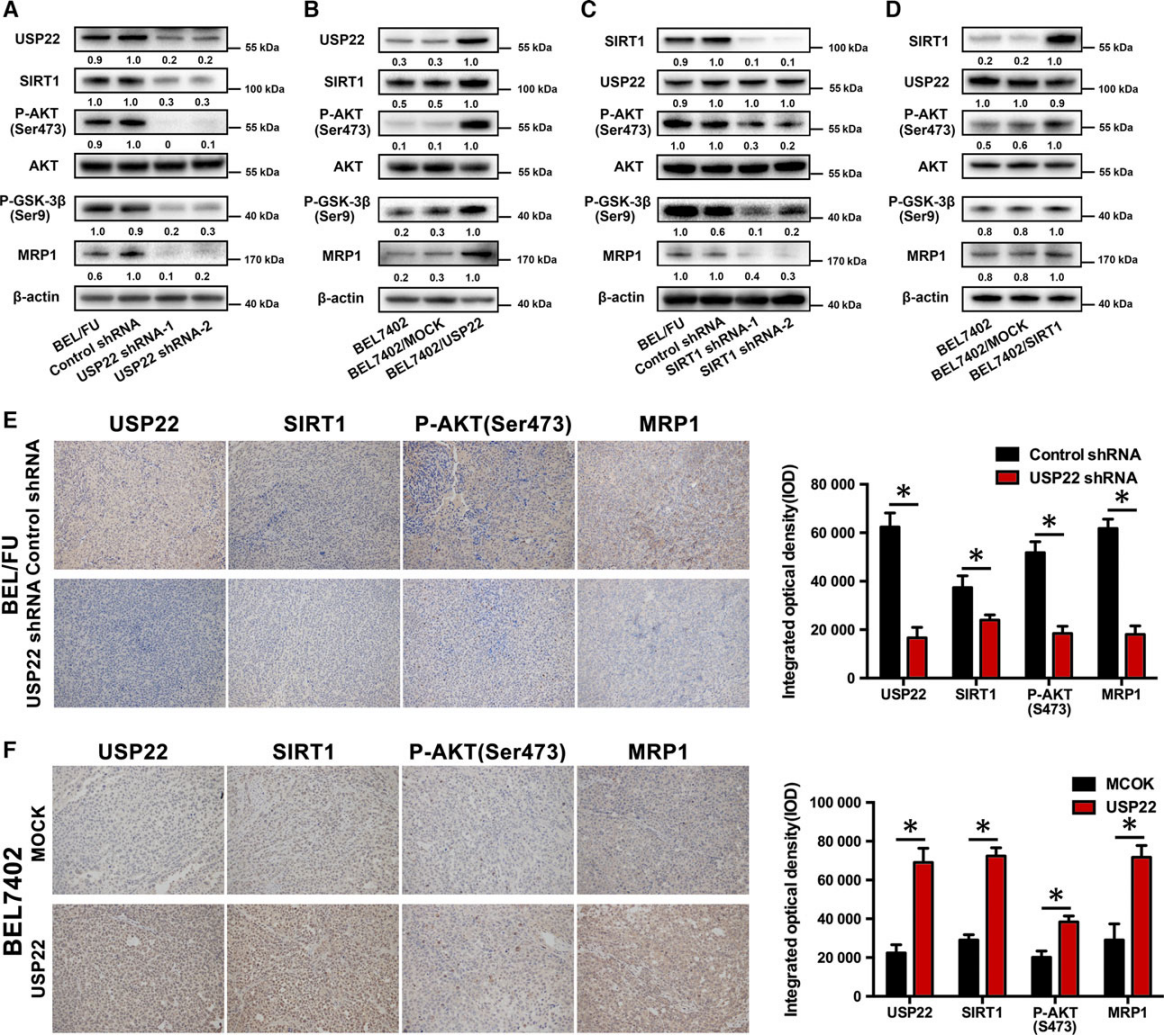
Regulation among USP22, SIRT1, and the AKT/MRP1 pathway. (A,B) After knockdown or overexpression of USP22 in BEL/FU or BEL7402 cells, USP22, SIRT1, phosphorylated AKT, AKT, phosphorylated GSK‐3β, and MRP1 were monitored using western blot analysis. (C,D) After knockdown or overexpression of SIRT1 in BEL/FU or BEL7402 cells, USP22, SIRT1, phosphorylated AKT, AKT, phosphorylated GSK‐3β, and MRP1 were monitored using western blot analysis. Band intensities were semiquantified using image lab 5.0 software and normalized with AKT or β‐actin. Values are represented as the means under the bands. (E,F) The expression of USP22, SIRT1, phosphorylated AKT, and MRP1 in xenograft tumors (derived from BEL/FU control shRNA, BEL/FU USP22 shRNA, BEL7402/mock, or BEL7402/USP22 cells) was detected by IHC. The data were quantified and are represented as the means ± SD (*P < 0.05).

**Figure 5:**
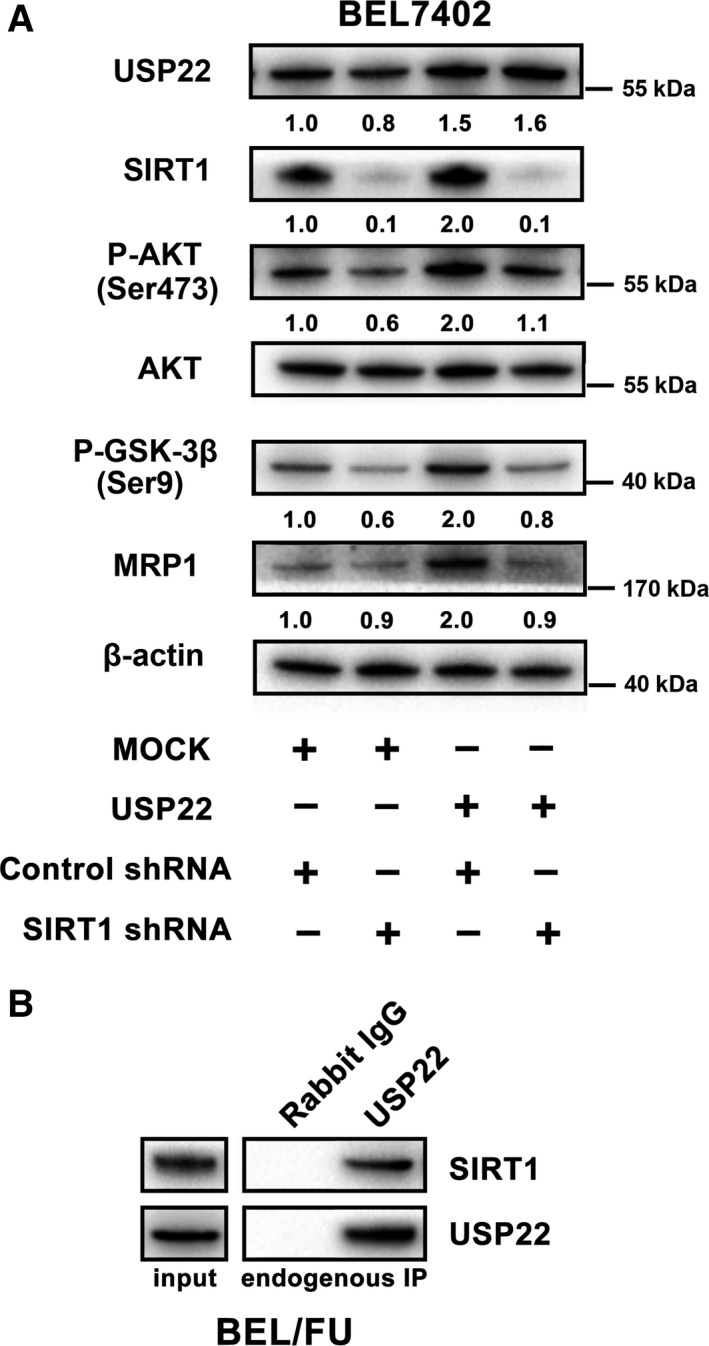
Deficiency of SIRT1 partially attenuates USP22‐induced activation of the AKT/MRP1 pathway in BEL7402 cells. (A) AKT/MRP1 pathway expression was monitored by western blot analysis in BEL7402 cells with USP22 overexpression and/or SIRT1 knockdown by shRNA. Values are represented as the means under the bands. (B) Co‐immunoprecipitation assays were used to verify the interaction between USP22 and SIRT1.

As shown in Fig. 4A, knockdown of USP22 by shRNA inhibited the AKT/MRP1 pathway compared with control shRNA cells and wild‐type cells. We observed remarkably decreased expression of phosphorylated AKT and phosphorylated GSK‐3β in BEL/FU USP22 shRNA cells. Similarly, overexpression of USP22 in BEL/7402 cells activated the AKT/MRP1 pathway (Fig. 4B). The downregulation of SIRT1 induced suppression of the AKT/MRP1 pathway in BEL/FU cells (Figs 4C and S2A), and the overexpression of SIRT1 activated the AKT/MRP1 pathway in BEL7402 cells (Fig. 4D). SIRT1 expression was positively regulated by USP22 (Fig. 4A,B), whereas SIRT1 knockdown or overexpression did not influence USP22 expression (Fig. 4C,D). Moreover, SIRT1 deficiency attenuated USP22‐induced activation of the AKT/MRP1 pathway in BEL7402 cells, suggesting that USP22 regulated the AKT/MRP1 pathway in a SIRT1‐dependent manner (Fig. 5A). USP22 directly interacts with SIRT1 based on the co‐immunoprecipitation assay (Fig. 5B).

In addition, we also evaluated the variation in SIRT1 and the AKT/MRP1 pathway in the xenografts of the nude mouse. Consistent with the *in vitro* results, SIRT1 and the AKT/MRP1 pathway were inhibited in BEL/FU USP22 shRNA cell‐derived xenografts and positively regulated in BEL/USP22 cell‐derived xenografts compared with control (Fig. 4E,F).

### 3.6 Modulation of SIRT1 in BEL/FU and BEL‐7402 cells regulates 5‐FU‐induced cell apoptosis and intracellular ADR concentrations

**Figure 6:**
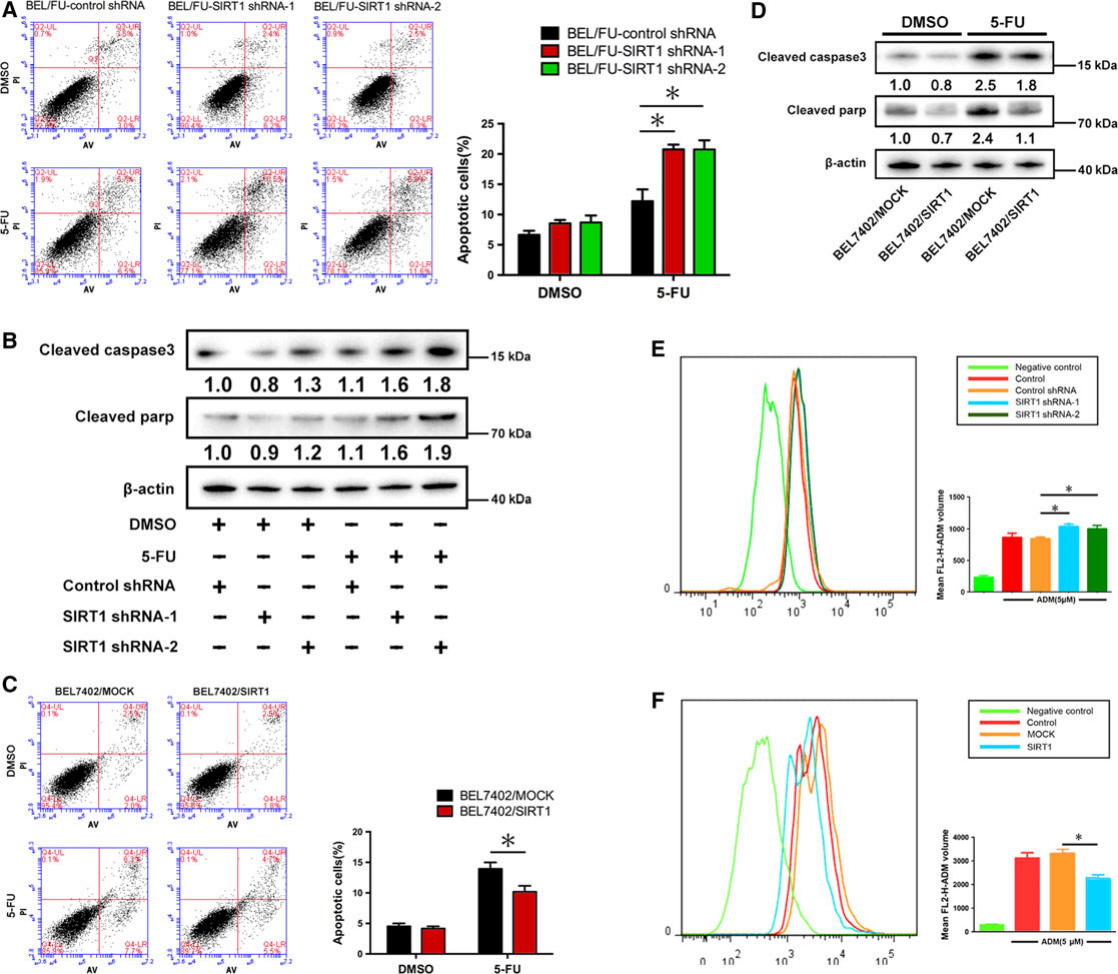
SIRT1 regulates cell apoptosis and intracellular ADR in BEL7402 and BEL/FU cells. (A,C) After knockdown or overexpression of SIRT1 in BEL/FU or BEL7402 cells, cells were examined using Annexin V/PI staining. Then, the distribution of apoptotic cells was measured by flow cytometric analysis. Apoptotic cell percentages are presented in the bar graph (*P < 0.05). (B,D) Cleaved caspase‐3 and cleaved parp were monitored using western blot analysis in BEL7402 and BEL/FU cells. Band intensities were semiquantified using image lab 5.0 software and normalized with AKT or β‐actin. Values are represented as the means under the bands. (E,F) Intracellular ADR was measured by flow cytometric analysis. Mean FL2‐H‐ADM volumes are presented in the bar graph as means ± SD from three independent experiments (*P < 0.05).

We next tested the effect of SIRT1 on 5‐FU‐induced cell apoptosis and intracellular ADR concentration. Constitutively suppressed SIRT1 caused a significant increase in apoptotic cells after 5‐FU treatment compared with the control cells in BEL/FU SIRT1 shRNA cells (Fig. 6A). Inhibition of SIRT1 stimulated the expression of cleaved caspase‐3 and cleaved parp (Fig. 6B) in BEL/FU cells treated with 5‐FU. Similarly, the apoptotic BEL‐7402/SIRT1 cells decreased upon treatment with 5‐FU (Fig. 6C). Cleaved caspase‐3 and cleaved parp were downregulated in BEL7402/SIRT1 cells (Fig. 6D). As shown in Fig. 6E,F, the concentration of intracellular ADR was increased in BEL/FU SIRT1 shRNA cells, and overexpression of SIRT1 reduced the intracellular ADR concentration in BEL7402 cells.

In conclusion, similar to USP22, SIRT1 has a remarkable influence on 5‐FU‐induced cell apoptosis and intracellular ADR concentration. These results confirmed the role of SIRT1 in USP22‐induced MDR in HCC cells.

### 3.7 Inhibition of the AKT signaling pathway suppresses the expression of MRP1 and promotes 5‐FU‐induced apoptosis in HCC cells

**Figure 7:**
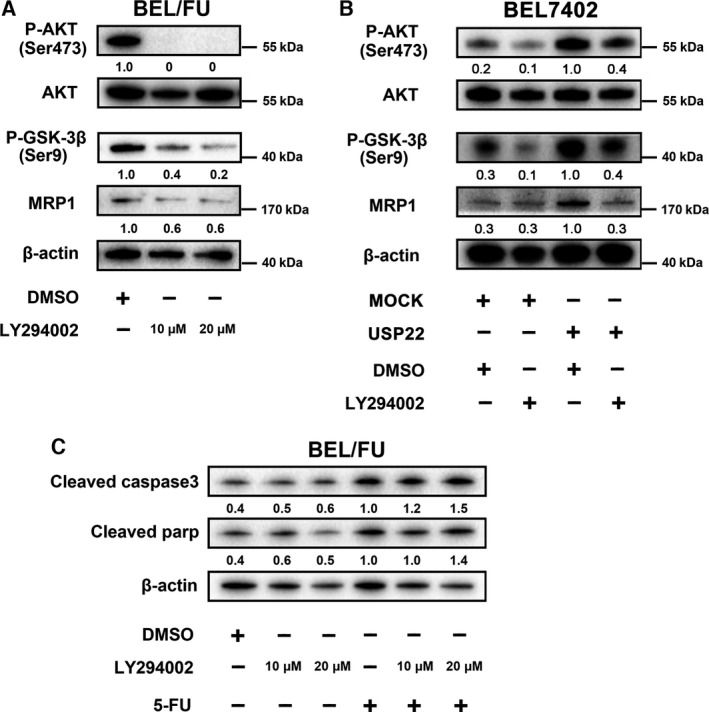
Inhibition of the AKT pathway suppresses MRP1 expression and promotes 5‐FU‐induced apoptosis in BEL/FU cells. (A) LY294002 (10 μm, 20 μm) was used to treat BEL/FU cells, and phosphorylated AKT, AKT, phosphorylated GSK‐3β, and MRP1 were monitored using western blot analysis. (B) AKT/MRP1 pathway expression was monitored by western blot analysis in BEL7402 cells with USP22 overexpression and/or LY294002. (C) Cleaved caspase‐3 and cleaved parp were monitored using western blot analysis in cells treated with LY294002 and/or 5‐FU. Band intensities were semiquantified using image lab 5.0 software and normalized with AKT or β‐actin. Values are represented as the means under the bands.

As aberrant expression of USP22 led to changes in the AKT pathway and MRP1, we tested whether MRP1 expression was regulated by AKT. Inhibition of the AKT pathway by LY294002 (10 μm, 20 μm) efficiently suppressed MRP1 expression in BEL/FU cells in both mRNA and protein levels (Figs 7A and S2B). LY294002 (10 μm) also suppressed USP22‐induced high expression of MRP1 via inhibition of AKT pathway in BEL7402 cells (Fig. 7B). Both of these findings indicated that USP22 upregulated MRP1 depending on the AKT pathway. In addition, cleaved caspase‐3 and cleaved parp were upregulated after BEL/FU cells treated with 5‐FU were incubated with LY294002 (Fig. 7C).

### 3.8 USP22 and MRP1 expressions are positively correlated in HCC tissues

**Figure 8:**
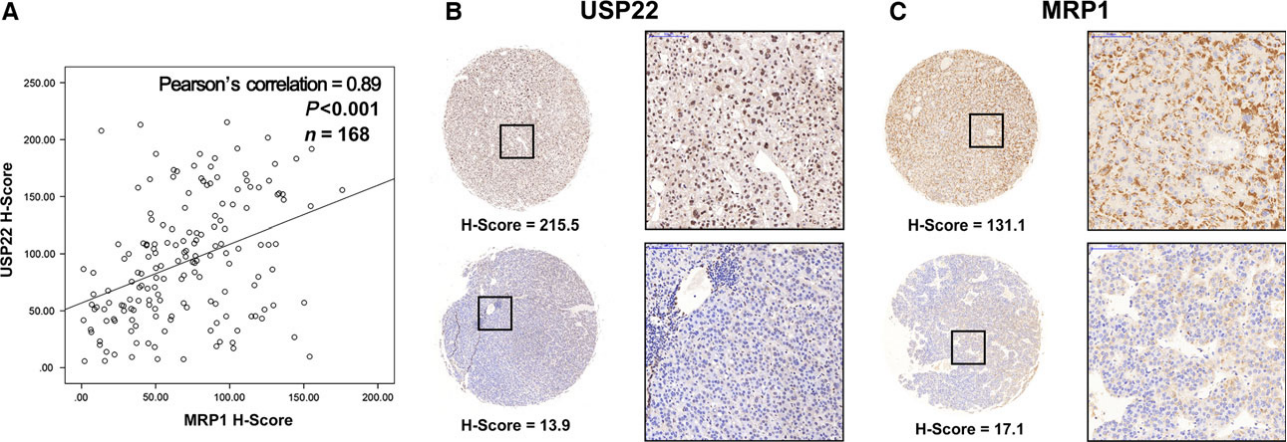
Correlation between USP22 and MRP1 protein expression in 168 HCC samples. (A) Positive correlation between USP22 (*y*‐axis) and MRP1 (*x*‐axis) H‐scores by immunohistochemistry (*P* < 0.001, Pearson's correlation = 0.89). (B) USP22 expression detected by immunohistochemical staining of the primary tumor in two patients diagnosed with HCC. (C) MRP1 expression detected by immunohistochemical staining of the primary tumor in two patients diagnosed with HCC.

Finally, USP22 and MRP1 expressions were detected in 168 HCC samples by immunohistochemical staining. Upon evaluation of H‐scores, a high positive correlation between USP22 and MRP1 was observed (*P *< 0.001, Pearson's correlation = 0.89) (Fig. 8B), further confirming the relationship between USP22 and MRP1.

## 4 Discussion

**Figure 9:**
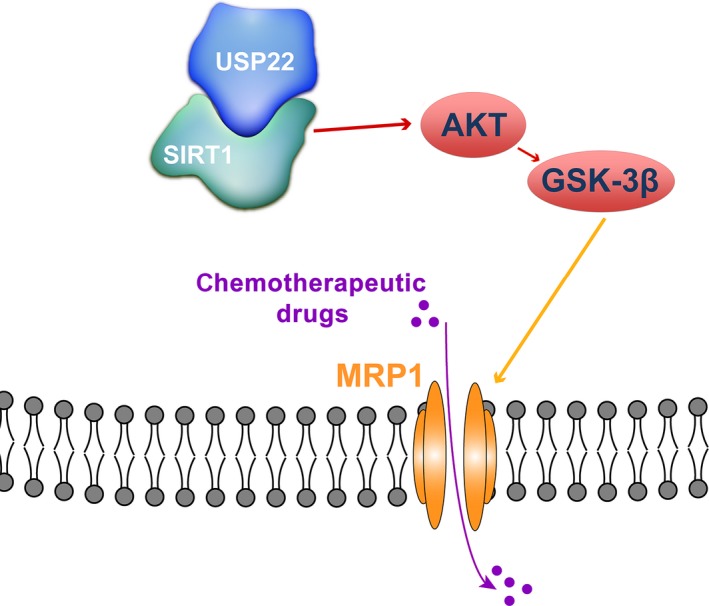
Proposed mechanisms responding to USP22‐mediated MDR in HCC. USP22 directly interacted with SIRT1 and then upregulated MRP1 through the activation of AKT/GSK‐3β pathway, which promoted the efflux of chemotherapeutic drugs of HCC cells.

Multidrug resistance is a multifactorial phenomenon and is the major obstacle in the successful and effective chemotherapeutic treatment for HCC. Elucidation of the mechanism of MDR will help to identify effective targets to reverse MDR of HCC in clinical settings. In this study, we demonstrated that USP22 is significant for the MDR of HCC cells *in vitro* and *in vivo* and deeply explored the mechanism underlying this phenomenon (Fig. 9).

First, we defined SIRT1 as a significant mediator in USP22‐driven MDR in HCC. Given that SIRT1 is deubiquitinated by USP22 and stabilized at the protein level (Armour *et al*., 2013; Lin *et al*., 2012) and previous studies have reported that SIRT1 could negatively influence the chemosensitivity of HCC cells (Chen *et al*., 2012), our results supported the notion that USP22 increases SIRT1 protein levels in HCC cells. Interestingly, we found that modulation of USP22 or SIRT1 could influence the intracellular ADR concentration, which might partly bridge the induction of MDR by USP22 and SIRT1 in HCC cells. Subsequently, using qPCR array analysis, we found that knockdown of USP22 could drastically inhibit the expression of MRP1, but not P‐gp, in BEL/FU cells. Overexpression of drug efflux transporters belonging to adenosine triphosphate‐dependent binding cassette (ABC) protein family is often responsible for the MDR of cancer (Kathawala *et al*., 2015; Wolking *et al*., 2015). The data of PCR array revealed some other interesting findings such as the downregulation of ABCC5 and CYP2D6, which regulates the drug metabolism in cell. We think it is valuable for us to investigate the relationship between USP22 and ABCC5 or CYP2D6. However, both MRP1, also known as ABCC1, and P‐gp, also known as ABCB1, play crucial roles in MDR of HCC (Ling *et al*., 2012b; Qian *et al*., 2015). Thus, we demonstrated that silencing of USP22 did not influence the protein level of P‐gp and further explored how USP22 regulated the expression of MRP1 and whether the process was dependent on SIRT1. We will demonstrate the relationship between USP22 and ABCC5 or CYP2D6 in our future studies.

The AKT signaling pathway controls the expression and function of numerous proteins, including MRP1, driving cancer cell MDR (Li *et al*., 2010; Wang *et al*., 2016b). Several studies have revealed the relationship between USP22 and the AKT signaling pathway (Cheng *et al*., 2013; Liu *et al*., 2012; Zhou *et al*., 2016; Zhuang *et al*., 2015). Therefore, the active status of the AKT signaling pathway was detected under the modulation of USP22 in HCC cells in the present study. We observed that USP22 could positively regulate the AKT pathway in a SIRT1‐dependent manner. The PI3K/AKT inhibitor (LY294002) could effectively suppress the expression of MRP1 and promote 5‐FU‐induced apoptosis in BEL/FU cells. Collectively, USP22 might deubiquitinate SIRT1 and subsequently activate the AKT pathway, increasing the expression of MRP1 to induce MDR in HCC cells. Several studies have described the regulation between SIRT1 and the AKT pathway (Pinton *et al*., 2013; Wang *et al*., 2016a). SIRT1 could deacetylate AKT and promote its activation (Sundaresan *et al*., 2011). Our results demonstrated that SIRT1 could promote the AKT activation, but not increased the expression level of AKT in HCC cells, which were consistent with the previous findings. The exact modification of the AKT pathway by SIRT1 in HCC cells needs further demonstration.

Moreover, we also identified a high expression correlation between USP22 and MRP1 in tumor samples from 168 HCC patients, which further confirmed the role of USP22 in the induction of MDR in HCC and supported the molecular mechanism we previously investigated. We will evaluate the clinical significance of combined USP22 and MRP1 detection in HCC tissues in predicting the survival rate or chemotherapy response in our future study.

Taken together, the present study found that USP22 can promote the MDR in HCC cells via activating the SIRT1/AKT/MRP1 pathway. USP22 might be a potential target, through which the MDR of HCC in clinical setting could be reversed. Given that SIRT1 is a promising regulator of metabolism and autophagy (Bellet *et al*., 2016; Ou *et al*., 2014), which are also significant for MDR in cancer cells (Bhattacharya *et al*., 2016), it is important to further explore the mechanism of USP22/SIRT1‐induced MDR in HCC cells. Moreover, an increasing number of USP22 substrates were discovered (Melo‐Cardenas *et al*., 2016). One of them is cyclin B1 (Lin *et al*., 2015). Owing to its relationship with the chemotherapeutic resistance of several types of human cancers (Glinsky, 2005), cyclin B1 may also mediate the USP22‐induced MDR in HCC cells, which is valuable for future exploration.

## Author contributions

SZ and XX conceived and coordinated the project. SL and JL designed the research study. SL, HX, QS, HD, and LD performed experiments and acquisition of data. SL, XW, PS, LZ, and JL analyzed and interpreted data. SL and JL wrote the paper and critically reviewed the manuscript. All authors read and approved the final version of the manuscript.

## Supporting information


**Fig. S1.** (A) Cancer drug resistance PCR array analysis in BEL/FU cells transfected with control shRNA or USP22 shRNA. A The gene list was presented as BEL/FU USP22 shRNA vs BEL/FU control shRNA. (B) Western blot assay was used to demonstrated that P‐gp (encoded by ABCB1) was not varied in BEL/FU cells with downregulation of USP22.

**Fig. S2.** MRP‐1 mRNA expression in BEL/FU cells with SIRT1 downregulation and AKT inactivation.
