# Supplementary figures and images for: USP22 mediates the multidrug resistance of hepatocellular carcinoma via the SIRT1/AKT/MRP1 signaling pathway
Source: Mol Oncol. 2017 May 11;11(6):682–95. doi: 10.1002/1878-0261.12067 (PMC5467492; doi:10.1002/1878-0261.12067)

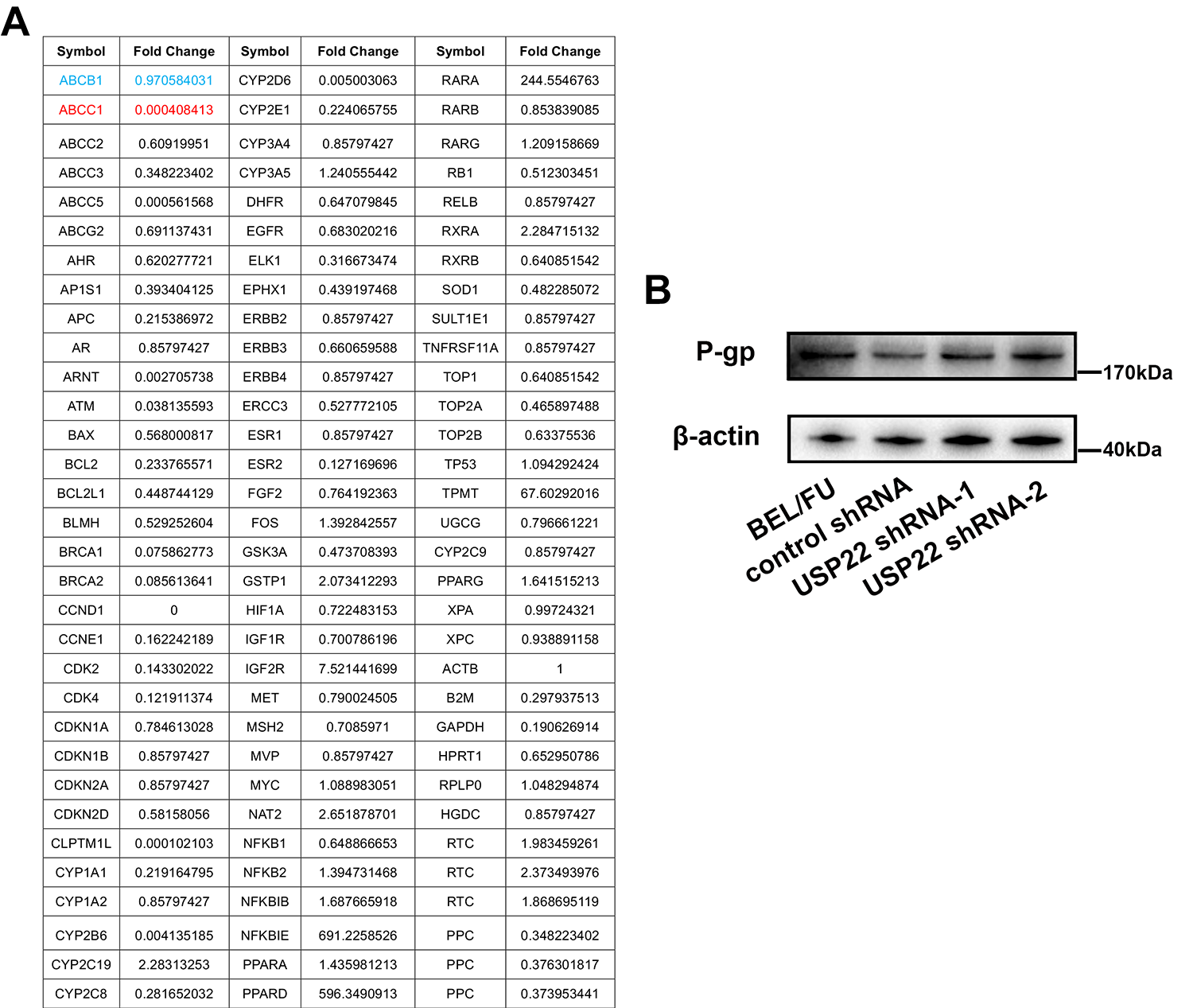

Supplement: Supplementary file 1 — Fig. S1. (A) Cancer drug resistance PCR array analysis in BEL/FU cells transfected with control shRNA or USP22 shRNA. A The gene list was presented as BEL/FU USP22 shRNA vs BEL/FU control shRNA. (B) Western blot assay was used to demonstrated that P‐gp (encoded by ABCB1) was not varied in BEL/FU cells with downregulation of USP22. [file MOL2-11-682-s001.tif]

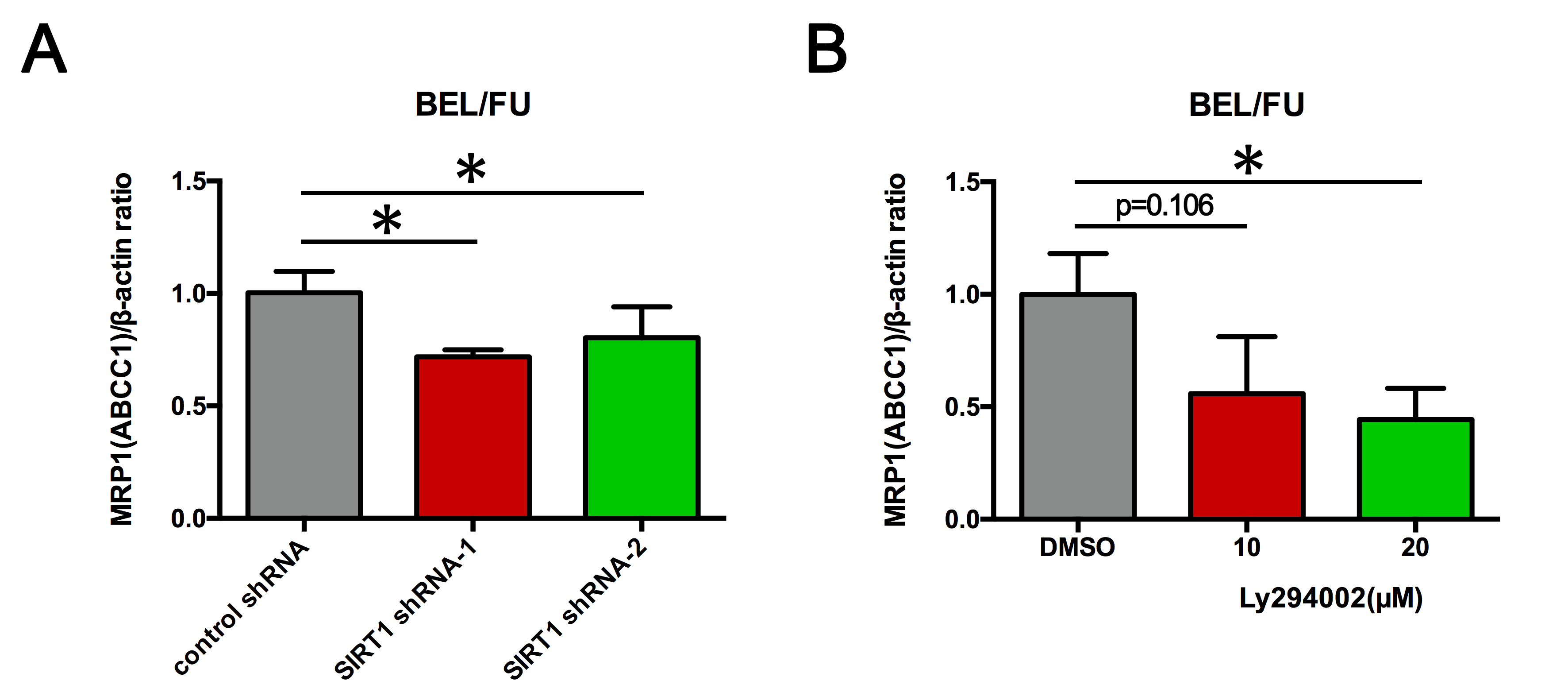

Supplement: Supplementary file 2 — Fig. S2. MRP‐1 mRNA expression in BEL/FU cells with SIRT1 downregulation and AKT inactivation. [file MOL2-11-682-s002.tif]
